# Gender, Sexual Orientation, and Workplace Incivility: Who Is Most Targeted and Who Is Most Harmed?

**DOI:** 10.3389/fpsyg.2016.00565

**Published:** 2016-05-02

**Authors:** Lauren Zurbrügg, Kathi N. Miner

**Affiliations:** ^1^Department of Psychology, Texas A&M University, College StationTX, USA; ^2^Department of Psychology and Women’s and Gender Studies Program, Texas A&M University, College StationTX, USA

**Keywords:** workplace incivilty, gender, sexual orientation, minority stress, intersectionality, occupational well-being

## Abstract

Scholars have proposed that interpersonal workplace discrimination toward members of oppressed social groups has become covert and subtle rather than overt and explicit and that such experiences lead to negative outcomes for targets. The present study examined this proposition by examining experiences and consequences of workplace incivility—a seemingly harmless form of interpersonal maltreatment—based on gender, sexual orientation, and their intersection. A sample of 1,300 academic faculty (52% male, 86% White) participated in an online survey study assessing their experiences of workplace incivility, job stress, job satisfaction, job identity centrality, and demographics. Results showed that sexual minority women reported the highest levels of workplace incivility. Findings also revealed that women reported lower job satisfaction than men and that heterosexuals reported higher job stress and lower job identity centrality than sexual minorities with higher levels of incivility. Thus, sexual minority status buffered the negative effects of incivility for sexual minorities. These findings point to the resiliency of sexual minorities in the face of interpersonal stressors at work.

## Introduction

Organizations have become more inclusive and tolerant of diversity (Thomas, 2012; Mor Barak, 2013), including enacting formal policies against workplace discrimination. However, research suggests that discrimination in organizations remains pervasive and that policies are often unenforced (Dipboye and Halverson, 2004; Goldman et al., 2006). For example, the U.S. Equal Employment Opportunity Commission (2015) received 88,778 discrimination charges during the 2014 fiscal year with an average of 90,445 yearly charges over the last decade. Discrimination may endure in organizations because it is more subtle than it was thirty or more years ago (Deitch et al., 2003; Dipboye and Halverson, 2004; Cortina, 2008; Jones et al., 2013). Moreover, covert forms of discrimination allow employees to continue to engage in discriminatory behavior while maintaining an unbiased image and evading punishment (Cortina, 2008; Sue, 2010).

One form of subtle discrimination, workplace incivility, had begun to receive considerable attention. Andersson and Pearson (1999) defined workplace incivility as rude and discourteous behavior in violation of workplace norms for mutual respect. Examples of incivility in work contexts include interruptions, excluding someone from professional camaraderie, and addressing a coworker inappropriately. A majority of workers cite incivility as being both common and a major issue in their work lives (Pearson et al., 2000; Pearson and Porath, 2009). Moreover, research shows that such behavior can interfere with the occupational well-being of employees who are targeted (Pearson et al., 2000; Cortina et al., 2001; Estes and Wang, 2008; Lim et al., 2008; Miner et al., 2012, 2014).

There remain critical gaps in the workplace incivility literature, however. For example, little is known about *who* is most at risk for being targeted with incivility at work. Incivility theorists (e.g., Cortina, 2008) propose that individuals in low-status social groups—such as women and sexual minorities—may be especially likely to experience uncivil treatment, as such behavior allows instigators a means to discriminate against individuals with low social status and power in a way that remains inconspicuous and maintains social dominance. Women and sexual minorities have been historically excluded from power and status in society (DiTomaso et al., 2007; Sue, 2010; Connell, 2014) and, further, empirical research has repeatedly documented that they are targets of overt and sometimes extreme forms of mistreatment, such as harassment and violence, due to their lower social status (Salin and Hoel, 2013; Smith et al., 2013; Holland and Cortina, 2016). We propose that these individuals may be frequent targets of incivility as well. We further propose that women and sexual minorities are the most harmed when targeted with workplace incivility because of their minority status (Meyer, 1995). In the coming sections, we build arguments for how and why status should affect experiences and outcomes of incivility for women and sexual minorities in work organizations.

### Who Is Most Targeted with Workplace Incivility?

Cortina’s (2008) theory of selective incivility was the first to frame incivility within the context of interpersonal discrimination. In contrast to formal discrimination, which is characterized by overtly discriminatory words or deeds, interpersonal discrimination is conveyed through subtle actions but may still represent more formal negative attitudes (Hebl et al., 2002; Sue, 2010). Cortina (2008) argues that targets of such behavior, namely women and racial minorities, are chosen in a systematic rather than unbiased manner and may be especially likely targets for workplace incivility because of their social group membership. Because uncivil interpersonal behaviors are seemingly harmless, perpetrators can mask their discriminatory attitudes toward women and people of color behind these acts and leave their personal image intact. Supporting selective incivility theory (Cortina, 2008), research shows that women and racial minorities are especially likely to experience uncivil treatment at work (Cortina et al., 2001, 2002, 2013; Settles and O’Connor, 2014).

Experiences of workplace incivility related to other status characteristics, such as sexual orientation, has received less attention. Scholars (Waldo, 1999; Ragins and Wiethoff, 2005; Sue, 2010) have argued that the workplace discrimination suffered by sexual minorities is also likely to be subtle and covert due to the nature of heterosexism and homophobia, which is often implicit. However, we could not identify even one study examining experiences of workplace incivility for sexual minorities. Research in other areas (e.g., education, cultural studies) suggests that sexual minorities may be at risk for experiencing subtle maltreatment. For example, Woodford et al. (2012) found that sexual minority college students were more likely to experience incivility at school compared to their heterosexual counterparts and Tomsen and Markwell (2009) found that sexual minorities reported experiences of threat and incivility during, and especially after, LGBT-based public events. In the microaggressions (i.e., discrimination in the form of verbal, behavioral, and environmental slights and indignities toward oppressed groups; Sue, 2010) literature, a number of qualitative (e.g., focus group) studies have documented sexual minorities’ experiences of subtle discrimination in the context of the university (Nadal et al., 2011; Platt and Lenzen, 2013), community (Nadal et al., 2011; Sarno and Wright, 2013; Bostwick and Hequembourg, 2014), and psychotherapy (Shelton and Delgado-Romero, 2011). We extend this past research by examining sexual minorities’ experiences of subtle negative treatment in the context of the workplace.

An additional critical omission from the workplace incivility literature is the extent to which multiple low-status group memberships intersect to affect experiences of incivility at work. Theories of intersectionality (McCall, 2005; Cole, 2009) purport that to fully understand individuals’ social-identity based experiences we must examine how different identities intersect to frame those experiences. Moreover, scholars (e.g., Acker, 2006; Holgate et al., 2006; Özbilgin et al., 2011) have emphasized the importance of applying the intersectionality approach to work environments. However, to date, the use of an intersectional lens to examine employees’ experiences of workplace incivility is rare. That is, little attention has been given to how experiences of uncivil treatment at work vary as a function of the intersection of different social categories. Indeed, the majority of findings to date are based on only one social identity—gender—without examining how other social identities may combine with gender to affect workers’ experiences of incivility. The one exception is the work of Cortina et al. (2013) who reported that gender and race interacted to affect experiences of incivility such that individuals holding multiple low-status social identities (i.e., women of color) reported the worst uncivil treatment. Research in the microaggressions literature also suggests that social identities intersect (e.g., race and sexual orientation, gender and religion, race and social class) to affect the experience of subtle slights and indignities in higher education and in the community (Balsam et al., 2011; Morales, 2014; Nadal et al., 2015).

Previous research lends support to the notion that low-status individuals are targeted more often with workplace incivility than majority-group members and that those holding multiple low-status identities are especially at risk. Corroborating and extending this past research, the present study examines the extent to which women and sexual minorities experience incivility at work, both as independent and interactive categories.

### Who Is Most Harmed by Workplace Incivility?

Workplace incivility has been conceptualized as a type of chronic stressor (Cortina et al., 2001; Lim et al., 2008). Chronic stressors differ from acute stressors in that they occur over an extended period of time and have ambiguous onsets and offsets (Hepburn et al., 1997). Lazarus and Folkman (1984) referred to these types of daily, persistent stressors as “daily hassles.” Such hassles are conceptualized as ongoing aggravations that occur as a part of life’s everyday roles, such as that of employee. These daily stressors, while low impact in the short term, accumulate to create deleterious work environments that can lead to well-being detriments for targeted individuals. In fact, they can be even more damaging to well-being than more dramatic life events (Kanner et al., 1981; DeLongis et al., 1982). This is because chronic stressors can accumulate to produce an additive “wear and tear” effect on victims through repeated exposure.

Consistent with theory, research has documented the negative well-being consequences of workplace incivility for those who are targeted, including increased job stress (Lim and Cortina, 2005; Kern and Grandey, 2009) and reduced job satisfaction (Cortina et al., 2001; Lim and Cortina, 2005; Lim et al., 2008; Miner et al., 2012). However, which individuals experience the worst outcomes as a result of incivility has yet to be fully investigated. We propose that the effects of incivility may not be equal across targets and that low-status individuals may be the most harmed by uncivil treatment. We also propose that workplace incivility affects job-related outcomes not yet assessed by researchers, such as employees’ identification with their job. We advance previous research by examining the extent to which individuals holding low-status social identities are differentially negatively affected by workplace incivility and assess a novel outcome of incivility: job identity centrality.

Minority stress theory is especially useful for understanding why low-status individuals might be likely to experience worse outcomes than high-status individuals when exposed to workplace incivility. The term “minority stress” was coined by Brooks (1981) and has been defined as “the stress experienced from being the member of a minority group that is marginalized and oppressed.” According to Brooks (1981), the stress that results from being a member of a minority group slowly accumulates over time leading to changes in the individual’s ability to process information and approach the world, such that the individual may not be able to effectively cope when faced with stressors. Brooks (1981) developed the concept of minority stress from her study of lesbian women, and Meyer (1995), who developed minority stress theory, studied the experiences of gay men. Therefore, these concepts are especially relevant to the mistreatment experiences and outcomes of sexual minorities.

In line with minority stress theory (Meyer, 1995), research shows that even seemingly minor acts of workplace discrimination can result in acute negative reactions for individuals who are members of marginalized minority groups. For example, research shows that women report more work withdrawal compared to men when they work in contexts tolerant of workplace incivility (Loi et al., 2015). Using an experimental paradigm, Woodzicka and LaFrance (2005) found that women showed worse performance during a job interview when the interviewer engaged in subtle incidents of harassment compared to women who did not have a harassing interviewer. Rospenda et al. (2009) found that experiences of workplace harassment and discrimination predicted problem drinking and mental health detriments and Lapierre et al. (2005) reported that non-sexual workplace aggression related to lower job satisfaction; these relationships were particularly pronounced for women. Research also shows that perceived discrimination relates to more psychological distress and job dissatisfaction for White and Black female professional employees; interestingly, the relationship between discrimination and distress were especially pronounced for White women, suggesting the intersection of social identities affects outcomes associated with subtle workplace mistreatment (Maddox, 2013).

Research also shows that workplace stressors based on sexual orientation (e.g., discrimination, unsupportive interactions) relate to heightened psychological distress (Smith and Ingram, 2004; Velez et al., 2013) and lowered job satisfaction (Velez et al., 2013) for sexual minority employees. Waldo (1999) found that experiences of workplace heterosexist discrimination were associated with lowered psychological and physical health and with heightened job withdrawal and job dissatisfaction among sexual minorities. Using an intersectional lens, Rabelo and Cortina (2014) found that concomitant experiences of workplace gender and sexual orientation based harassment were associated with greater job burnout and lower job satisfaction in a sample of sexual minority employees in higher education. Perceived workplace sex and sexual orientation discrimination has also been linked to work withdrawal and, in turn, lateness, absenteeism, and intentions to quit (Volpone and Avery, 2013).

### The Present Study

The purpose of the present study is to examine the extent to which demographic characteristics associated with societal power and status make employees vulnerable to experiencing incivility at work. Specifically, we investigate whether employees in two low-status social groups—women and sexual minorities—report more frequent uncivil workplace experiences and show more pronounced negative outcomes with higher levels of incivility compared to their higher-status counterparts—males and heterosexuals. Importantly, we also investigate whether employees who hold multiple low-status identities are most targeted with and harmed by incivility at work.

This study’s contributions are fourfold. First, we extend the literature on workplace incivility by examining two dimensions of status: gender and sexual orientation. Second, we investigate these dimensions not only independently, but also at their intersection. Third, we seek to address the fundamental question of whether low-status individuals, especially those who belong to multiple minority groups, experience a greater frequency of uncivil work behaviors compared to members of one or more dominant social groups. Fourth, we investigate how low-status individuals’ occupational well-being may be affected by receiving uncivil treatment. In light of the call for and importance of real-life implications for research on discrimination (e.g., Fiske, 2000), these contributions are especially important in that they allow researchers to begin to examine how uncivil treatment affects low-status employees.

### Research Hypotheses

Based on the past research and theory in this area, we hypothesize the following:

#### Hypothesis 1

Women report experiencing more workplace incivility compared to men (a) and sexual minorities report experiencing more workplace incivility compared to heterosexuals (b).

#### Hypothesis 2

Gender and sexual orientation interact to predict experiences of incivility such that sexual minority women report the highest levels of incivility.

#### Hypothesis 3

Women report worse outcomes (i.e., higher job stress and lower job satisfaction and job identity centrality) compared to men (a) and sexual minorities report worse outcomes compared to heterosexuals (b) with higher levels of incivility.

#### Hypothesis 4

Gender and sexual orientation interact to predict the severity of outcomes related to workplace incivility such that sexual minority women report the worst outcomes with higher levels of incivility.

## Materials and Methods

### Participants

Participants for this study included a nationwide sample of academic law professors. The final sample (*N* = 1,300) was 52% male (*n* = 652) and 86% White (*n* = 1,107). Sixty-six participants (2.2%) reported their ethnicity as Black, African, or African–American, 29 (2.3%) as Hispanic or Hispanic–American, 28 (2.2%) as Asian–American or Pacific Islander, 13 (1%) as Native-American or Alaskan Native, and 9 (0.7%) as Middle Eastern, Arab, or Arab-American; 36 participants (2.8%) did not reported their ethnicity. Participants’ age ranged from 27 to 80 years (*M* = 50.65, *SD* = 10.05). Employment with their present law school (*M* = 13.29, *SD* = 8.85) and years teaching law (*M* = 15.51, *SD* = 8.72) both ranged from less than 1 year to more than 30 years. The number of faculty in their department ranged from 19 to 91 (*M* = 44.08, *SD* = 16.09).

Participants were asked to indicate their sexual orientation using the following choices based on Kinsey et al.’s (1948, 1953) research showing that sexual orientation is more accurately represented by a continuum than a heterosexual/homosexual binary: completely homosexual, lesbian, or gay (*n* = 65, 5%); mostly homosexual, lesbian, or gay (*n* = 17, 1.3%); bisexual (*n* = 14, 1.1%); mostly heterosexual (*n* = 46, 3.6%); and completely heterosexual (*n* = 1,140, 88.5%). Eighteen (1%) participants did not indicate their sexual orientation and were therefore excluded from analyses involving this variable. The first four categories were combined to comprise sexual minority status (*n* = 145), consistent with previous empirical research (Silverschanz et al., 2008; Woodford et al., 2012); sexual minorities were coded as 0 and heterosexuals coded as 1.

### Procedure

In June 2004, an e-mail was sent to all members of the Association of American Law Schools (AALS) (*N* = 8,929) asking them to participate in a study examining “quality of life in law academia.” The e-mail contained a brief description of the study and a link to an online survey. The email also stated that participation was completely voluntary, that participants could skip any question, and that participants’ privacy would be protected. Completion of the survey served as consent to participate in the survey. Nine-hundred of the invitation e-mails were rejected due to e-mail filters or inaccurate e-mail addresses; thus, the total potential pool of participants was 8,029. Of these, 1,810 responded to the survey (a 23% response rate). Five-hundred and ten of these participants were excluded due to skipping more than 50% of the items on the survey.

### Instrumentation

The measures for the present study (i.e., experiences of workplace incivility, occupational well-being, and demographics) represent a subset of those included in the larger survey. Survey construction focused on minimizing response bias and utilizing valid and reliable measures. All items were scored such that higher values reflected higher levels of the underlying construct.

#### Workplace Incivility

Participants’ experiences of workplace incivility were assessed using the *Workplace Incivility Scale* (*WIS*; Cortina et al., 2001; Caza and Cortina, 2007). This scale measures the degree to which respondents perceived being a target of rude and disrespectful behavior at work. Instructions asked participants to indicate how often a coworker had instigated any of nine behaviors (e.g., “made jokes at your expense,” “made insulting or disrespectful remarks to you”) within the last year, using a response scale from 1 (*never)* to 4 (*frequently*). The *WIS* has been shown to be highly reliable (α = 0.89) and to have good convergent validity, as indicated by a significant negative correlation (*r* = −0.56, *p* < 0.001) with the *Perceptions of Fair Interpersonal Treatment* (*PFIT*) scale (Donovan et al., 1998; Cortina et al., 2001). A complete account of the development and validation of the *WIS* using a large employee sample is available in Cortina et al. (2001). Internal reliability for this measure in the present study was 0.85.

#### Occupational Well-Being

Participants’ occupational well-being was assessed with measures of job stress, job satisfaction, and job identity centrality. Job stress was measured with an abbreviated six-item version of Stanton et al.’s (2001) *Stress in General* (*SIG*) scale, a global measure of job stress with good convergent and discriminant validity. Items ask whether each of a list of adjectives (e.g., “hectic,” “tense,” “pressured”) is descriptive of the respondent’s job, using a “*no*,” “*?*,” “*yes*” response format. A complete account of the extensive development and validation of this measure with three diverse samples of workers is available in Stanton et al. (2001). Internal reliability for this measure in the present study was 0.82.

Job satisfaction was measured with the *Michigan Organizational Assessment Questionnaire* (*MOAQ*; Cammann et al., 1979, unpublished). Respondents indicated on a scale ranging from 1 (*strongly disagree)* to 7 *(strongly agree)* the extent to which each of three statements characterized their work: “All in all, I am satisfied with my job,” “In general, I like working here,” and “In general, I don’t like my job” (reverse coded). A full description of the development and validation of this measure is available in Cammann et al. (1979, unpublished) and Seashore et al. (1982). Recent meta-analytic analyses also indicate that the *MOAQ* is a reliable and construct-valid measure of job satisfaction (Bowling and Hammond, 2008). Internal reliability for this measure in the present study was 0.89.

Job identity centrality was measured with a revised version of the importance subscale of the *Collective Self-Esteem Scale* (Luhtanen and Crocker, 1992). Participants responded to five items assessing the extent to which being a member of their law school’s faculty was central to their identity using a 1 (*strongly disagree)* to 7 *(strongly agree)* response scale. Example items include “In general, being a member of the law faculty is an important part of my self-image” and “Overall, being a member of the law faculty has very little to do with how I feel about myself” (reverse-coded). Luhtanen and Crocker (1992) describe the full development and validation of this measure, demonstrating its strong psychometric properties in three separate studies. Internal reliability for this measure in the present study was 0.83.

#### Control Variables

We included four demographic variables as covariates in the analyses to help isolate the effects of the variables of interest. These control variables included organizational tenure, years teaching law, department size, and age.

## Results

All analyses were conducted using IBM Statistical Package for the Social Science (SPSS) Version 23 software (IBM Corp, 2014). **Table 1** displays the means, standard deviations, and intercorrelations for all study variables. Workplace incivility was positively correlated with job stress and negatively correlated with job satisfaction and job identity centrality. In addition, the covariates (organizational tenure, years teaching, department size, and age) were correlated with incivility and at least one of the outcomes, corroborating our decision to include them as covariates in the analyses.

**Table 1:** Means, standard deviations, and intercorrelations for all study variables.

Variable	*M*	*SD*	1	2	3	4	5	6	7
(1) Workplace Incivility	1.55	0.60							
(2) Job Satisfaction	5.76	1.35	–0.48^∗∗∗^						
(3) Job Stress	1.67	0.58	0.38^∗∗∗^	–0.46^∗∗∗^					
(4) Job Identity Centrality	4.53	1.30	–0.19^∗∗∗^	0.41^∗∗∗^	–0.15^∗∗∗∗^				
(5) Organization Tenure	13.29	8.85	–0.09^∗∗^	0.08^∗∗^	–0.16^∗∗∗^	0.15^∗∗∗^			
(6) Years Teaching	15.51	8.72	–0.10^∗∗∗^	0.09^∗∗^	–0.14^∗∗∗^	0.20^∗∗∗^	0.87^∗∗∗^		
(7) Age	50.65	10.05	–0.13^∗∗∗^	0.10^∗∗^	–0.12^∗∗∗^	0.13^∗∗∗^	0.70^∗∗∗^	0.76^∗∗∗^	
(8) Department Size	44.08	16.09	–0.08^∗∗^	0.06∗	0.00	0.01	0.03	0.06∗	0.07∗

*∗p < 0.05, ∗∗p < 0.01, ∗∗∗p < 0.001.*

**FIGURE 1:**
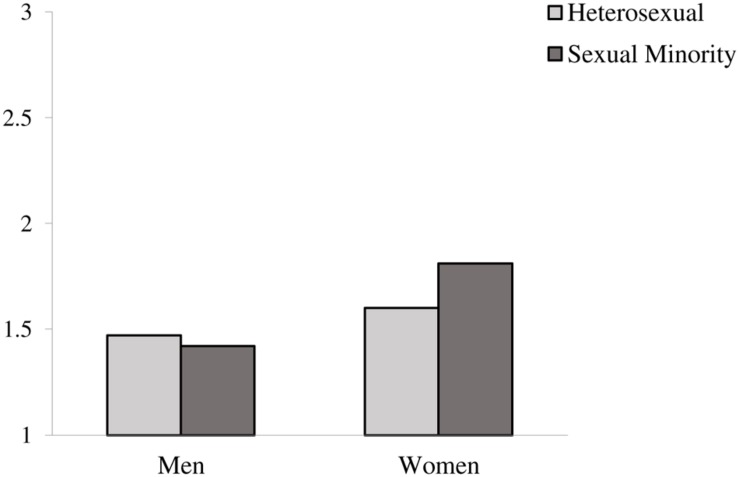
**Interaction of gender and sexual orientation on workplace incivility**.

Hypothesis 1 predicted that women would report experiencing more workplace incivility compared to men (a) and that sexual minorities would report experiencing more workplace incivility compared to heterosexuals (b). Hypothesis 2 predicted that gender and sexual orientation would interact to predict experiences of incivility such that sexual minority women would report the highest levels of incivility. We conducted an ANCOVA to test these hypotheses. Gender and sexual orientation were the predictor variables and workplace incivility was the outcome variable in the analyses. The ANCOVA yielded a main effect of gender, *F*(1,1103) = 17.83, *p* < 0.01, $\eta_{p}^{2}$ = 0.02 such that women (*M* = 1.63, *SD* = 0.62) reported higher levels of workplace incivility compared to men (*M* = 1.47, *SD* = 0.55). While in the expected direction, sexual minorities (*M* = 1.65, *SD* = 0.62) and heterosexuals (*M* = 1.53, *SD* = 0.58) reported comparable levels of workplace incivility, *F*(1,1103) = 1.86, *p* = 0.17. Thus, Hypothesis 1a was fully supported and Hypothesis 1b was not supported. The ANCOVA also revealed a gender × sexual orientation interaction on workplace incivility, *F*(1,1103) = 6.12, *p* < 0.05, $\eta_{p}^{2}$ = 0.01. Follow-up simple effects tests revealed that the effect of gender was significant both within sexual minorities [*F*(1,128) = 11.57, *p* < 0.01, $\eta_{p}^{2}$ = 0.09] and within heterosexuals [*F*(1,975) = 6.16, *p* < 0.05, $\eta_{p}^{2}$ = 0.01], and that the effect of sexual orientation was significant within women [*F*(1,542) = 7.50, *p* < 0.01, $\eta_{p}^{2}$ = 0.01], but not within men [*F*(1,561) = 0.59, *p* = 0.44]. Supporting Hypothesis 2, these tests revealed that the mean for sexual minority women (*M* = 1.81, *SD* = 0.65) differed significantly from that of heterosexual women, heterosexual men, and sexual minority men. In terms of the pattern of the means, heterosexual women reported the second highest mean (*M* = 1.60, *SD* = 0.61), followed by heterosexual men (*M* = 1.47, *SD* = 0.56), and sexual minority men, who reported the lowest levels of incivility (*M* = 1.42, *SD* = 0.51). The interaction between gender and sexual orientation on workplace incivility is displayed in **Figure 1.**

Hypothesis 3 predicted that women would report worse outcomes (i.e., higher job stress and lower job satisfaction and job identity centrality) compared to men (a) and that sexual minorities would report worse outcomes compared to heterosexuals (b) with higher levels of workplace incivility. Further, Hypothesis 4 predicted that gender and sexual orientation would interact such that sexual minority women would report the worst outcomes with higher levels of incivility. These hypotheses were tested via hierarchical moderated regression. Workplace incivility was the predictor, gender and sexual orientation were the moderators, and job stress, job satisfaction, and job identity centrality were the outcome variables in the analyses. To correct for multicollinearity, we centered the incivility variable before computing interaction terms multiplicatively. Organizational tenure, years teaching, department size, and age were again included as covariates. **Tables 2–4** summarize the results of these analyses.

**Table 2:** Hierarchical regression analysis examining gender and sexual orientation as moderators of incivility and job stress.

	Model 1			Model 2			Model 3			Model 4		
Predictors	*B*	*SE*	β	*B*	*SE*	β	*B*	*SE*	β	*B*	*SE*	β
Organizational tenure	–0.01	0.00	–0.12†	0.00	0.00	–0.10†	–0.01	0.00	–0.10†	0.01	0.00	–0.10†
Years teaching	0.00	0.00	–0.03	0.00	0.00	–0.02	0.00	0.00	0.00	0.00	0.00	–0.01
Department size	0.00	0.00	0.00	0.00	0.00	0.00	0.00	0.00	0.02	0.00	0.00	0.02
Age	0.00	0.00	–0.02	0.00	0.00	0.02	0.00	0.00	0.04	0.00	0.00	0.04
Workplace incivility				0.36	0.03	0.36∗∗∗	0.33	0.05	0.33∗∗∗	0.35	0.05	0.36***
Gender				–0.22	0.02	–0.19∗∗∗	–0.26	0.05	–0.22∗∗∗	–0.26	0.05	–0.22***
Sexual orientation				–0.02	0.02	–0.02	–0.04	0.03	–0.04	–0.03	0.03	–0.03
Incivility × Gender							–0.08	0.06	–0.06	–0.17	0.09	–0.12†
Incivility × Orientation							0.09	0.04	0.09∗	0.05	0.05	0.06
Gender × Orientation							0.04	0.05	0.04	0.04	0.05	0.04
Incivility × Gender × Orientation										0.11	0.09	0.08
*R*^2^		0.03			0.20			0.21			0.21	
*F* for change in *R*^2^		7.14∗∗∗			82.15∗∗∗			2.10†			1.59	


†*p < 0.10, ∗p < 0.05, ∗∗∗p < 0.001.*

**Table 3:** Hierarchical regression analysis examining gender and sexual orientation as moderators of incivility and job satisfaction.

	Model 1			Model 2			Model 3			Model 4		
Predictors	*B*	*SE*	β	*B*	*SE*	β	*B*	*SE*	β	*B*	*SE*	β
Organizational tenure	0.00	0.01	0.00	0.00	0.01	–0.01	0.00	0.01	–0.01	0.00	0.01	–0.01
Years teaching	0.01	0.01	0.06	0.01	0.01	0.04	0.01	0.01	0.03	0.01	0.01	0.03
Department size	0.00	0.00	0.04	0.00	0.00	0.02	0.00	0.00	0.00	0.00	0.00	0.02
Age	0.01	0.01	0.04	0.00	0.01	–0.02	0.00	0.01	–0.01	0.00	0.01	–0.01
Workplace incivility				–1.07	0.06	–0.46***	–1.18	0.11	–0.52***	–1.22	0.11	–0.53***
Gender				0.21	0.07	0.08**	0.16	0.12	0.06	0.16	0.12	0.06
Sexual orientation				0.13	0.06	0.06*	0.09	0.08	0.04	0.08	0.08	0.04
Incivility × Gender							0.40	0.13	0.12**	0.53	0.20	0.16**
Incivility × Orientation							–0.10	0.09	–0.04	–0.05	0.11	–0.02
Gender × Orientation							0.08	0.12	0.04	0.08	0.12	0.03
Incivility × Gender × Orientation										–0.16	0.20	–0.05
*R*^2^		0.01			0.24			0.25			0.25	
*F* for change in *R*^2^		2.70∗		110.30∗∗∗				3.90∗			1.63	

*∗p < 0.05, ∗∗p < 0.01, ∗∗∗p < 0.001.*

**Table 4:** Hierarchical regression analysis examining gender and sexual orientation as moderators of incivility and job identity centrality.

	Model 1			Model *2*			Model 3			Model 4		
Predictors	*B*	*SE*	β	*B*	*SE*	β	*B*	*SE*	β	*B*	*SE*	β
Organizational tenure	–0.01	0.01	–0.08	–0.01	0.01	–0.09	–0.01	0.01	–0.09	–0.01	0.01	0.09
Years teaching	0.05	0.01	0.31***	0.05	0.01	0.31***	0.05	0.01	0.31***	0.05	0.01	0.31***
Department size	0.00	0.00	0.00	0.00	0.00	–0.01	0.00	0.00	–0.01	0.00	0.00	–0.01
Age	–0.01	0.01	–0.05	–0.01	0.01	–0.08†	–0.01	0.01	–0.08*	–0.01	0.01	0.09*
Workplace incivility				–0.35	0.07	–0.16***	–0.07	0.01	–0.03	–0.09	0.12	–0.04
Gender				0.28	0.08	0.10**	0.28	0.13	0.11*	0.30	0.13	0.11*
Sexual orientation				–0.01	0.00	0.00	0.02	0.08	0.01	0.01	0.08	0.01
Incivility × Gender							–0.25	0.13	–0.08	–0.15	0.21	–0.05
Incivility × Orientation							–0.22	0.10	–0.10*	–0.19	0.12	–0.08
Gender × Orientation							–0.01	0.12	0.00	–0.01	0.12	0.00
Incivility × Gender × Orientation										–0.11	0.21	–0.04
*R*^2^		0.04			0.08			0.09			0.09	
*F* for change in *R*^2^		12.44^∗∗^			14.66∗∗∗			3.44∗			0.29	

†*p < 0.10, ∗p < 0.05, ∗∗p < 0.01, ∗∗∗p < 0.001.*

**FIGURE 2:**
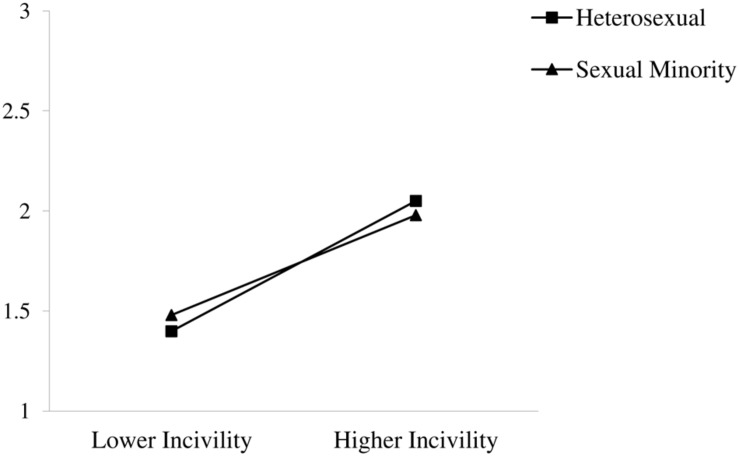
**Interaction of workplace incivility and sexual orientation on job stress**.

As shown in **Table 2**, there were significant main effects of workplace incivility and gender on job stress. The more participants reported experiencing incivility at work the higher their job stress. In addition, women reported more job stress than men. The main effect of incivility was qualified by an incivility × sexual orientation interaction on job stress. The interaction was graphed (see **Figure 2**) and simple slope analyses were conducted to examine the nature of this relationship. Results showed that although sexual minorities reported greater job stress with higher levels of incivility (β = 0.30, *SE* = 0.08, *p* < 0.01), this relationship was more pronounced for heterosexuals *(*β = 0.39, *SE* = 0.03, *p* < 0.001). The hypothesized incivility × sexual orientation and incivility × gender × sexual orientation interactions were not significant. As such, for job stress, Hypotheses 3a, 3b, and 4 were not supported.

**FIGURE 3:**
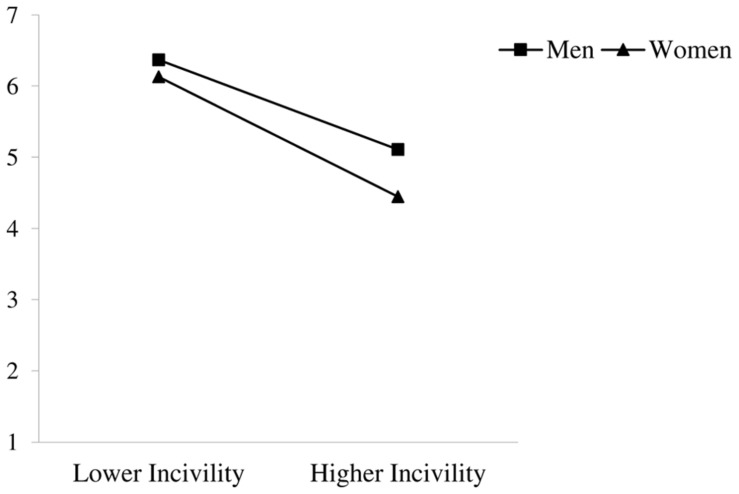
**Interaction of workplace incivility and gender on job satisfaction**.

There were also main effects of workplace incivility, gender, and sexual orientation on job satisfaction (see **Table 3**). Participants reported lower levels of job satisfaction with greater experiences of incivility at work, and men and heterosexuals reported higher job satisfaction than did women and sexual minorities, respectively. The main effects of incivility and gender were qualified by an incivility × gender interaction on job satisfaction, which is displayed in **Figure 3.** Simple slope analyses revealed that although men reported lower job satisfaction with higher levels of incivility *(*β = −0.39, *SE* = 0.09, *p* < 0.001), this relationship was especially pronounced for women *(*β = −0.53, *SE* = 0.09, *p* < 0.001). The hypothesized incivility × sexual orientation interaction was not significant. Thus, Hypothesis 3a was fully supported and Hypothesis 3b was not supported for job satisfaction. The results also did not reveal the hypothesized incivility × gender × sexual orientation interaction; therefore, Hypothesis 4 was not supported for job satisfaction.

**FIGURE 4:**
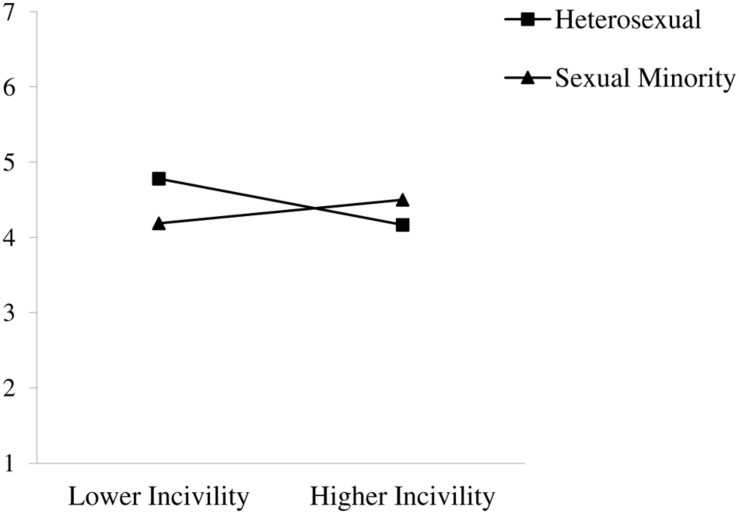
**Interaction of workplace incivility and sexual orientation on job identity centrality**.

Finally, results revealed main effects of workplace incivility and gender on job identity centrality (see **Table 4**). Participants reported lower levels of identity centrality with greater experiences of incivility at work, and men reported higher centrality than did women. The main effect of incivility was qualified by an incivility × sexual orientation interaction on job identity centrality (see **Figure 4**). Simple slope analyses revealed that heterosexuals reported lower identity centrality with higher levels of incivility *(*β = −0.19, *SE* = 0.07, *p* < 0.001); this relationship was not significant for sexual minorities *(*β = 0.01, *SE* = 0.18, *ns*). The hypothesized incivility × gender and incivility × gender × sexual orientation interactions were not significant. As such, for job identity centrality, Hypotheses 3a, 3b, and 4 were not supported.

## Discussion

The present study examined the extent to which gender and sexual orientation make employees vulnerable to a subtle, seemingly innocuous from of discrimination: workplace incivility. We also examined how experiences of incivility at work relate to job stress, job satisfaction, and job identity centrality as a function of gender and sexual orientation. While there is a large body of literature documenting that members of low-status groups (e.g., women, sexual minorities) are at risk for high-intensity mistreatment at work (Salin and Hoel, 2013; Smith et al., 2013; Holland and Cortina, 2016), there has been far less research documenting the experiences of low-status individuals when targeted with subtle forms of mistreatment, such as workplace incivility. Further, while Cortina et al. (2001, 2002, 2013) have examined differences in the frequency of incivility experiences and the severity of outcomes by gender, no studies to our knowledge have investigated the incivility experiences of sexual minorities and only one study has examined experiences of incivility for those with multiple minority identities (e.g., women of color; Cortina et al., 2013). The present study addressed these lacunae in the literature by being the first to examine the workplace incivility experiences of sexual minorities; we also examined experiences of incivility for those holding multiple low-status identities (i.e., sexual minority women).

Selective incivility theory (Cortina, 2008) posits that individuals are not randomly chosen for uncivil treatment but, rather, that individuals with low-status identities are especially likely to be targeted. Further, minority stress theory (Brooks, 1981; Meyer, 1995) predicts that low-status individuals are especially negatively affected by such experiences because of the daily stress they face as a minority-group member. On the basis of these theories, we predicted that low-status employees (i.e., women and sexual minorities) would report higher frequencies of uncivil experiences and show more pronounced negative work outcomes with higher levels of incivility compared to their higher-status counterparts (i.e., men and heterosexuals). We also hypothesized those employees who hold multiple low-status identities (i.e., sexual minority women) would be most frequently targeted and most negatively affected by incivility.

Partially supporting our first hypothesis, women reported experiencing more incivility at work compared to men; however, sexual minorities did not report experiencing more incivility compared to heterosexuals. These findings are both consistent with and deviate from past research. Confirming past research (Cortina et al., 2001, 2002, 2013; Settles and O’Connor, 2014), our findings suggest that women are especially vulnerable to being treated uncivilly at work. However, refuting past research (Tomsen and Markwell, 2009; Woodford et al., 2012), sexual minorities (in general) did not differ from heterosexuals in the extent to which they experienced incivility.

Confirming our second hypothesis, sexual minority women reported the highest levels of incivility at work. This finding supports intersectionality theory (McCall, 2005; Cole, 2009) and previous findings in the microaggressions literature (e.g., Balsam et al., 2011; Nadal et al., 2015). Interestingly, sexual minority men reported the lowest levels of workplace incivility. One possibility for this finding may be that sexual minority men, even though they hold a low-status sexual identity, benefit from the social status and power that comes with being male which overrides their vulnerability to mistreatment based on their sexual orientation.

Consistent with minority stress theory (Brooks, 1981; Meyer, 1995), women reported lower job satisfaction than men with higher levels of workplace incivility. This finding extends those of Loi et al. (2015) who found that women reported more work withdrawal than men in work environments characterized by incivility. However, conflicting with minority stress theory (Brooks, 1981; Meyer, 1995), sexual minority women were not the most negatively affected by workplace incivility, even though they reported the highest levels of uncivil workplace behavior. Rather, results showed that heterosexuals were more negatively affected by being treated uncivilly at work. Specifically, findings revealed that heterosexuals reported higher job stress and lower job identity centrality with higher levels of incivility compared to sexual minorities. Thus, holding a lower-status sexual orientation seemed to buffer the negative effects of incivility for sexual minorities, contrary to our third and fourth hypotheses and past research on more extreme forms of mistreatment like heterosexist discrimination (e.g., Waldo, 1999; Smith and Ingram, 2004; Velez et al., 2013). It may be the case that sexual minorities have habituated to living in a society where they regularly experience subtle incivilities and have gained resilience in the face of such stressors thereby lessening the negative effects on their well-being (Sue, 2010; Hill and Gunderson, 2015; Meyer, 2015).

### Limitations and Future Research Directions

Although the present study addressed several important gaps in the workplace incivility literature, there are a number of limitations that should be acknowledged. An obvious limitation is the reliance on single-source self-report data, which could lead to common method bias (Podsakoff et al., 2012). When this bias occurs, relationships between constructs tend to be inflated, possibly leading to inaccurate conclusions. One indicator of common method bias is high correlations among the measures included in a study. That many of the relationships we assessed showed variability in effect sizes (i.e., low to moderate to high) suggests that common method bias was not a major concern in the present study. To address this issue in the future, researchers might consider collecting data from multiple sources, such as supervisors or close family members of the employee.

The categorization of sexual orientation is also a limitation. Indeed, the experiences of different sexual minorities may be unique with, for example, bisexual individuals having different mistreatment experiences than lesbians and gay men. Another important aspect of sexual identity that was not assessed in this study is “outness.” Outness is important because sexual orientation is a “hidden identity” and the extent to which one is mistreated likely depends on how “out” one is at work (Claire et al., 2005; Ragins, 2008; Gates, 2014; Prati and Pietrantoni, 2014). Although we did not assess the incivility experiences of bisexuals or as a function of outness, the present study takes a first step toward understanding the frequency and consequences of workplace incivility for sexual minorities, an important contribution to the literature. Even so, the assessment of outness and the examination of bisexuals’ experiences of workplace incivility are critical next steps for research in this area.

There are also likely other status-linked variables not assessed in the present study (e.g., race, disability, social class) that intersect with gender and/or sexual orientation to predict experiences of workplace incivility and the severity of outcomes. Selective incivility theory (Cortina, 2008) specifically proposes that women and racial minorities are likely targets of workplace incivility and research supports this proposition (Cortina et al., 2001, 2002, 2013; Settles and O’Connor, 2014). Preliminary research also suggests that gender and race interact to affect experiences of incivility (Cortina et al., 2013). As such, the inclusion of race as a key variable in future research seems especially germane to understanding how different social categories combine to affect employees’ experiences of incivility at work. We urge future research to examine race and other social identities in concert to better understand how incivility affects employees. In so doing, researchers can begin to identify social identities that are most and least vulnerable to workplace incivility and its consequences.

Finally, the findings of the present study may not be generalizable to other industries, occupations, and individuals with very different status characteristics. Indeed, the sample included in the present research was primarily composed of White, highly educated individuals in a unique job field (academia). Assessing the constructs of interest in a more diverse sample in terms of race, social class, age, disability, etc. may yield different results. Future research should select specific organizations and industries in which more diverse samples can be recruited in terms of ethnicity, sexual orientation, education, SES, industry, and job type.

### Implications for Practice

Based on the findings presented in the present paper, we offer several suggestions for curbing workplace incivility, especially toward women, sexual minorities, and other low-status employees. First, organizations should institute formal policies declaring their intolerance of interpersonal maltreatment, especially maltreatment targeted at particular social groups. For example, policies could specify that disrespectful, offensive behavior will not be accepted and that employees should treat one another with dignity, respect, and consideration (Mor Barak, 2013). By setting expectations and standards for respectful interpersonal treatment, organizations convey the importance of workplace interactions and provide guidance for everyday work conduct (Pearson and Porath, 2009).

Second, careful selection and training programs can also promote a more civil work environment (Pearson and Porath, 2009; Reio and Ghosh, 2009; Gedro and Wang, 2013; Leiter et al., 2015). For example, organizations could check potential employees’ references and past employment histories to screen for potential problems with interpersonal behavior. Organizations might also communicate to new employees the importance of respectful workplace behavior and ask what qualities they have that could contribute to such an environment. Training to enhance interpersonal skills and sensitivity to coworkers would also be beneficial. Such training could also provide employees with strategies to avoid and deal with disrespectful interactions. Githens (2011) proposes that the most transformative programs to combat incivility targeted toward minorities are those that both educate and take action by addressing unconscious biases and taking steps to create inclusive environments for all employees.

Finally, organizations should consider instituting Employee Assistance and Stress Management Programs to deal with the stresses associated with experiencing uncivil behavior at work (Giebels and Janssen, 2005; Richardson and Rothstein, 2008; Leiter et al., 2015). These programs can provide a variety of counseling, support services, and stress-reduction techniques to employees who are victims of negative workplace behavior. Given the frequency of incivility in the workplace, such programs may prove particularly beneficial for helping employees deal with consequences interpersonal mistreatment.

## Conclusion

This study advances the literature on subtle workplace discrimination by examining whether women and sexual minorities are more frequently targeted with and negatively affected by experiences of workplace incivility compared to their higher-status counterparts. Results suggest that sexual minority women are most targeted with workplace incivility compared to sexual minority men and heterosexual women and men, but that women (regardless of sexual minority status) and heterosexuals (regardless of gender) are most harmed by incivility experiences. Future research should explore the extent to which these findings replicate in other settings and assess additional low-status social identities both independently and simultaneously.

## Author Contributions

All authors listed, have made substantial, direct and intellectual contribution to the work, and approved it for publication.

## Conflict of Interest Statement

The authors declare that the research was conducted in the absence of any commercial or financial relationships that could be construed as a potential conflict of interest.

## References

[B1] Acker J. (2006). Inequality regimes: gender, class, and race in organizations. *Gender Soc.* 20 441–464. 10.1177/0891243206289499

[B2] Andersson L. M. Pearson C. M. (1999). Tit for tat? The spiraling effect of incivility in the workplace. *Acad. Manag. Rev.* 24 452–471. 10.2307/259136

[B3] Balsam K. F. Molina Y. Beadnell B. Simoni J. Walters K. (2011). Measuring multiple minority stress: the LGBT people of color microaggressions scale. *Cult. Divers Ethnic. Minor. Psychol.* 17 163–174. 10.1037/a0023244 PMC4059824 21604840

[B4] Bostwick W. Hequembourg A. (2014). ‘Just a little hint’: bisexual-specific microaggressions and their connection to epistemic injustices. *Cult. Health Sex.* 16 488–503. 10.1080/13691058.2014.889754 24666221

[B5] Bowling N. A. Hammond G. D. (2008). A meta-analytic examination of the construct validity of the michigan organizational assessment questionnaire job satisfaction subscale. *J. Vocat. Behav.* 73 63–77. 10.1016/j.jvb.2008.01.004

[B6] Brooks V. R. (1981). *Minority Stress and Lesbian Women.* Lexington, MA: D. C. Heath.

[B7] Caza B. B. Cortina L. M. (2007). From insult to injury: explaining the impact of incivility. *Basic Appl. Soc. Psychol.* 29 335–350. 10.1080/01973530701665108

[B8] Claire J. A. Beatty J. E. Maclean T. L. (2005). Out of sight but not out of mind: Managing invisible social identities in the workplace. *Acad. Manag. Rev.* 30 78–95. 10.5465/AMR.2005.15281431

[B9] Cole E. R. (2009). Intersectionality and research in psychology. *Am. Psychol.* 64 170–180. 10.1037/a0014564 19348518

[B10] Connell R. W. (2014). *Gender and Power: Society, the Person and Sexual Politics.* Hoboken, NJ: John Wiley & Sons.

[B11] IBM corporation (2014). *IBM SPSS Statistics for Windows, Version 23.0.* Armonk, NY: IBM Corp.

[B12] Cortina L. M. (2008). Unseen injustice: incivility as modern discrimination in organizations. *Acad. Manag. Rev.* 33 55–75. 10.5465/AMR.2008.27745097

[B13] Cortina L. M. Kabat-Farr D. Leskinen E. A. Huerta M. Magley V. J. (2013). Selective incivility as modern discrimination in organizations: evidence and impact. *J. Manage.* 39 1579–1605.

[B14] Cortina L. M. Lonsway K. A. Magley V. J. Freeman L. V. Collinsworth L. L. Hunter M. (2002). What’s gender got to do with it? Incivility in the Federal Courts. *Law Soc. Inquiry* 27 235–270. 10.1111/j.1747-4469.2002.tb00804.x

[B15] Cortina L. M. Magley V. J. Williams J. H. Langhout R. D. (2001). Incivility in the workplace: incidence and impact. *J. Occup. Health Psychol.* 6 64–80. 10.1037/1076-8998.6.1.64 11199258

[B16] Deitch E. A. Barsky A. Butz R. M. Brief A. P. Chan S. Bradley J. C. (2003). Subtle yet significant: the existence and impact of everyday racial discrimination in the workplace. *Hum. Relat.* 56 1299–1324. 10.1177/00187267035611002

[B17] DeLongis A. Coyne J. C. Dakof G. Folkamn S. Lazarus R. S. (1982). Relationship of daily hassles, uplifts, and major life events to health status. *Health Psychol.* 1 119–136. 10.1037/0278-6133.1.2.119

[B18] Dipboye R. L. Halverson S. K. (2004). “Subtle (and not so subtle) discrimination in organizations,” in *The Dark Side of Organizational Behavior*, eds Griffin R. W. O’Leary-Kelly A. M. (San Francisco: Jossey-Bass), 131–158.

[B19] DiTomaso N. Post C. Parks-Yancy R. (2007). Workforce diversity and inequality: power, status, and numbers. *Annu. Rev. Sociol.* 33 473–501. 10.1146/annurev.soc.33.040406.131805

[B20] Donovan M. A. Drasgow F. Munson L. J. (1998). The perceptions of fair interpersonal treatment scale: development and validation of a measure of interpersonal treatment in the workplace. *J. Appl. Psychol.* 83 683–692. 10.1037/0021-9010.83.5.683 9806012

[B21] Estes B. Wang J. (2008). Workplace incivility: impacts on individual and organizational performance. *Hum. Resour. Dev. Rev.* 7 218–240. 10.1177/1534484308315565

[B22] Fiske S. T. (2000). Stereotyping, prejudice, and discrimination at the seam between centuries: evolution, culture, mind, and brain. *Eur. J. Soc. Psychol.* 20 299–322. 10.1002/(SICI)1099-0992(200005/06)30:3<299::AID-EJSP2>3.0.CO;2-F

[B23] Gates T. G. (2014). Assessing the relationship between outness at work and stigma consciousness among LGB workers in the Midwest and the resulting implications for counselors. *Couns. Psychol. Q.* 27 264–276. 10.1080/09515070.2014.886998

[B24] Gedro J. Wang J. (2013). Creating civil and respectful organizations through the scholar-practitioner bridge. *Adv. Dev. Hum. Resour.* 15 284–295. 10.1177/1523422313488062

[B25] Giebels E. Janssen O. (2005). Conflict stress and reduced well-being at work: the buffering effect of third-party help. *Eur. J. Work Organ. Psychol.* 14 137–155. 10.1080/13594320444000236

[B26] Githens R. P. (2011). Diversity and incivility: toward an action-oriented approach. *Adv. Dev. Hum. Resour.* 13 40–53.

[B27] Goldman B. M. Gutek B. A. Stein J. H. Lewis K. (2006). Employment discrimination in organizations: antecedents and consequences. *J. Manage.* 32 786–830.

[B28] Hebl M. R. Foster J. B. Mannix L. M. Dovidio J. F. (2002). Formal and interpersonal discrimination: a field study of bias toward homosexual applicants. *Pers. Soc. Psychol. Bull.* 28 815–825. 10.1177/0146167202289010

[B29] Hepburn C. G. Loughlin C. A. Barling J. (1997). “Coping with chronic work stress,” in *Coping with Chronic Stress*, ed. Gottlieb B. H. (New York, NY: Plenum Press), 343–366.

[B30] Hill C. A. Gunderson C. J. (2015). Resilience of lesbian, gay, and bisexual individuals in relation to social environment, personal characteristics, and emotion regulation strategies. *Psychol. Sex. Orient. Gender Diver.* 2 232–252. 10.1037/sgd0000129

[B31] Holgate J. Hebson G. McBride A. (2006). Why gender and ‘difference’ matters: a critical appraisal of industrial relations research. *Indust. Relat. J.* 37 310–328. 10.1111/j.1468-2338.2006.00406.x

[B32] Holland K. J. Cortina L. M. (2016). “Sexual harassment: undermining the well-being of working women,” in *Handbook on Well-Being of Working Women*, (Berlin: Springer), 83–101.

[B33] Jones K. P. Peddie C. I. Gilrane V. L. King E. B. Gray A. L. (2013). Not so subtle: A meta-analytic investigation of the correlates of subtle and overt discrimination. *J. Manag.* 1–26. 10.1177/0149206313506466

[B34] Kanner A. D. Coyne J. C. Schaefer C. Lazarus R. S. (1981). Comparisons of two modes of stress management: daily hassles and uplifts versus major life events. *J. Behav. Med.* 4 1–39. 10.1007/BF00844845 7288876

[B35] Kern J. H. Grandey A. A. (2009). Customer incivility as a social stressor: the role of race and racial identity for service employees. *J. Occup. Health Psychol.* 14 46–57. 10.1037/a0012684 19210046

[B36] Kinsey A. C. Pomeroy W. B. Martin C. E. (1948). *Sexual Behavior in the Human Male.* Philadelphia: Saunders.

[B37] Kinsey A. C. Pomeroy W. B. Martin C. E. Gebhard P. H. (1953). *Sexual Behavior in the Human Female.* Philadelphia: Saunders.

[B38] Lapierre L. M. Spector P. E. Leck J. D. (2005). Sexual versus nonsexual workplace aggression and victims’ overall job satisfaction: a meta-analysis. *J. Occup. Health Psychol.* 10 155–169. 10.1037/1076-8998.10.2.155 15826225

[B39] Lazarus R. S. Folkman S. (1984). *Stress, Appraisal, and Coping.* New York, NY: Springer.

[B40] Leiter M. P. Peck E. Gumuchian S. (2015). “Workplace incivility and its implications for well-being,” in *Mistreatment in Organizations*, (Bingley: Emerald Group Publishing Limited), 107–135.

[B41] Lim S. Cortina L. M. (2005). Interpersonal mistreatment in the workplace: the interface and impact of general incivility and sexual harassment. *J. Appl. Psychol.* 90 483–496. 10.1037/0021-9010.90.3.483 15910144

[B42] Lim S. Cortina L. M. Magley V. J. (2008). Personal and workgroup incivility: impact on work and health outcomes. *J. Appl. Psychol.* 93 95–107. 10.1037/0021-9010.93.1.95 18211138

[B43] Loi N. M. Loh J. M. Hine D. W. (2015). Don’t rock the boat: the moderating role of gender in the relationship between workplace incivility and work withdrawal. *J. Manag. Dev.* 34 169–186. 10.1108/JMD-12-2012-0152

[B44] Luhtanen R. Crocker J. (1992). A collective self-esteem scale: self-evaluation of one’s social identity. *Pers. Soc. Psychol. Bull.* 18 302–318. 10.1177/0146167292183006

[B45] Maddox T. (2013). Professional women’s well-being: the role of discrimination and occupational characteristics. *Women Health* 53 706–729. 10.1080/03630242.2013.822455 24093451 PMC3806220

[B46] McCall L. (2005). The complexity of intersectionality. *Signs* 30 1771–1800. 10.1086/426800

[B47] Meyer I. H. (1995). Minority stress and mental health in gay men. *J. Health Sci. Soc. Behav.* 36 38–56. 10.2307/2137286 7738327

[B48] Meyer I. H. (2015). Resilience in the study of minority stress and health of sexual and gender minorities. *Psychol. Sex. Orient. Gender Diver.* 2 209–213. 10.1037/sgd0000132

[B49] Miner K. N. Settles I. H. Brady C. Pratt-Hyatt J. (2012). Experiencing incivility in organizations: the buffering effects of emotional and organizational support. *J. Appl. Soc. Psychol.* 42 340–372. 10.1111/j.1559-1816.2011.00891.x

[B50] Miner K. N. Smittick A. Pesonen A. Seigel M. L. Clark E. (2014). Does being a mom help or hurt? The relationship between workplace incivility and job outcomes as a function of motherhood status. *J. Occup. Health Psychol.* 19 60–73. 10.1037/a0034936 24447221

[B51] Mor Barak E. M. (2013). *Managing Diversity: Toward a Globally Inclusive Workplace.* Thousand Oaks: Sage.

[B52] Morales E. M. (2014). Intersectional impact: black students and race, gender and class microaggressions in higher education. *Race Gender Class* 21 48–66.

[B53] Nadal K. L. Davidoff K. C. Davis L. S. Wong Y. Marshall D. McKenzie V. (2015). A qualitative approach to intersectional microaggressions: understanding influences of race, ethnicity, gender, sexuality, and religion. *Q. Psychol.* 2 147–163. 10.1037/qup0000026

[B54] Nadal K. L. Issa M. A. Leon J. Meterko V. Wideman M. Wong Y. (2011). Sexual orientation microaggressions: “Death by a thousand cuts” for lesbian, gay, and bisexual youth. *J. LGBT Youth* 8 234–259. 10.1080/19361653.2011.584204

[B55] Özbilgin M. F. Beauregard T. A. Tatli A. Bell M. P. (2011). Work–life, diversity and intersectionality: a critical review and research agenda. *Int. J. Manag. Rev.* 13 177–198. 10.1111/j.1468-2370.2010.00291.x

[B56] Pearson C. M. Andersson L. M. Porath C. L. (2000). Assessing and attacking workplace incivility. *Organ. Dyn.* 29 123–137. 10.1016/S0090-2616(00)00019-X

[B57] Pearson C. M. Porath C. L. (2009). *The Cost of Bad Behavior: How Incivility Damages Your Business and What You Can Do About It.* New York: Penguin Group.

[B58] Platt L. F. Lenzen A. L. (2013). Sexual orientation microaggressions and the experience of sexual minorities. *J. Homosexual.* 60 1011–1034. 10.1080/00918369.2013.774878 23808348

[B59] Podsakoff P. M. MacKenzie S. B. Podsakoff N. P. (2012). Sources of method bias in social science research and recommendations on how to control it. *Annu. Rev. Psychol.* 63 539–569. 10.1146/annurev-psych-120710-100452 21838546

[B60] Prati G. Pietrantoni L. (2014). Coming out and job satisfaction: a moderated mediation model. *Career Dev. Q.* 62 358–371. 10.1002/j.2161-0045.2014.00088.x

[B61] Rabelo V. C. Cortina L. M. (2014). Two sides of the same coin: gender harassment and heterosexist harassment in LGBQ work lives. *Law Hum. Behav.* 38 378–391. 10.1037/lhb0000087 24933169

[B62] Ragins B. R. (2008). Disclosure disconnects: antecedents and consequences of disclosing invisible stigmas across life domains. *Acad. Manag. Rev.* 33 194–215. 10.5465/AMR.2008.27752724

[B63] Ragins B. R. Wiethoff C. (2005). “Understanding heterosexism at work: the straight problem,” in *Discrimination at Work: The Psychological and Organizational Bases*, eds Dipboye R. L. Colella A. (Mahwah, NJ: Lawrence Erlbaum Associates).

[B64] Reio T. G. Ghosh R. (2009). Antecedents and outcomes of workplace incivility: Implications for human resource development research and practice. *Hum. Resour. Dev. Q.* 20 237–264. 10.1002/hrdq.20020

[B65] Richardson K. M. Rothstein H. R. (2008). Effects of occupational stress management intervention programs: a meta-analysis. *J. Occup. Health Psychol.* 13 69–93. 10.1037/1076-8998.13.1.69 18211170

[B66] Rospenda K. M. Richman J. A. Shannon C. A. (2009). Prevalence and mental health correlates of harassment and discrimination in the workplace: results from a national study. *J. Interpers. Violence* 24 819–843. 10.1177/0886260508317182 18463311 PMC3979593

[B67] Salin D. Hoel H. (2013). Workplace bullying as a gendered phenomenon. *J. Manag. Psychol.* 28 235–251. 10.1108/02683941311321187

[B68] Sarno E. Wright A. J. (2013). Homonegative microaggressions and identity in bisexual men and women. *J. Bisex.* 13 63–81. 10.1080/15299716.2013.756677

[B69] Seashore S. E. Lawler E. E. Mirvis P. Cammann C. (eds). (1982). *Observing and Measuring Organizational Change: A Guide to Field Practice.* New York: Wiley.

[B70] Settles I. H. O’Connor R. C. (2014). Incivility at academic conferences: gender differences and the mediating role of climate. *Sex Roles* 71 71–82. 10.1007/s11199-014-0355-y

[B71] Shelton K. Delgado-Romero E. A. (2011). Sexual orientation microaggressions: the experience of lesbian, gay, bisexual, and queer clients in psychotherapy. *J. Couns Psychol.* 58:210 10.1037/a0022251 21463031

[B72] Silverschanz P. Cortina L. M. Konik J. Magley V. (2008). Slurs, snubs, and queer jokes: incidence and impact of heterosexist harassment in academia. *Sex Roles* 58 179–191. 10.1007/s11199-007-9329-7

[B73] Smith I. P. Oades L. McCarthy G. (2013). The Australian corporate closet, why it’s still so full: a review of incidence rates for sexual orientation discrimination and gender identity discrimination in the workplace. *Gay Lesbian Issues Psychol. Rev.* 9 51–63.

[B74] Smith N. G. Ingram K. M. (2004). Workplace heterosexism and adjustment among lesbian, gay, and bisexual individuals: the role of unsupportive social interactions. *J. Couns. Psychol.* 51 57–67. 10.1037/0022-0167.51.1.57

[B75] Stanton J. M. Balzer W. K. Smith P. C. Parra L. F. Ironson G. (2001). A general measure of work stress: the stress in general scale. *Educ. Psychol. Measur.* 61 866–888. 10.1177/00131640121971455

[B76] Sue D. W. (2010). *Microaggressions in Everyday Life: Race, Gender, and Sexual Orientation.* Hoboken, NJ: John Wiley & Sons.

[B77] Thomas K. M. (ed.). (2012). *Diversity Resistance in Organizations.* New York: Psychology Press.

[B78] Tomsen S. Markwell K. (2009). Violence, cultural display and the suspension of sexual prejudice. *Sexual. Cult.* 13 201–217. 10.1007/s12119-009-9054-1

[B79] U.S. Equal Employment Opportunity Commission. (2015). *Charge Statistics FY 1997 Through 2014.* Available at: http://www.eeoc.gov/eeoc/statistics/enforcement/charges.cfm [Accessed November 23 2015].

[B80] Velez B. L. Moradi B. Brewster M. E. (2013). Testing the tenets of minority stress theory in workplace contexts. *J. Couns. Psychol.* 60 532–542. 10.1037/a0033346 23815632

[B81] Volpone S. D. Avery D. R. (2013). It’s self defense: how perceived discrimination promotes employee withdrawal. *J. Occup. Health Psychol.* 18 430–448. 10.1037/a0034016 24099162

[B82] Waldo C. R. (1999). Working in a majority context: a structural model of heterosexism as minority stress in the workplace. *J. Couns. Psychol.* 46 218–232. 10.1037/0022-0167.46.2.218

[B83] Woodford M. R. Krentzman A. R. Gattis M. N. (2012). Alcohol and drug use among sexual minority college students and their heterosexual counterparts: the effects of experiencing and witnessing incivility and hostility on campus. *Subst. Abuse Rehabil.* 3 11–23. 10.2147/SAR.S26347 24474863 PMC3886646

[B84] Woodzicka J. A. LaFrance M. (2005). The effects of subtle sexual harassment on women’s performance in a job interview. *Sex Roles* 53 67–77. 10.1007/s11199-005-4279-4

